# Early Empirical Tuberculosis Treatment in HIV-Positive Patients Admitted to Hospital in South Africa: An Observational Cohort Study

**DOI:** 10.1093/ofid/ofab162

**Published:** 2021-03-31

**Authors:** Carolin Bresges, Douglas Wilson, Katherine Fielding, Elizabeth L Corbett, Fabrizia Del-Greco, Daniel Grint, Jurgens Peters, Ankur Gupta-Wright

**Affiliations:** 1 Global Health and Infection Department, Brighton and Sussex Medical School, Brighton, United Kingdom; 2 Clinical Research Department, London School of Hygiene and Tropical Medicine, London, United Kingdom; 3 Department of Internal Medicine, Edendale Hospital, University of KwaZulu-Natal, Pietermaritzburg, South Africa; 4 Department of Infectious Disease Epidemiology, London School of Hygiene and Tropical Medicine, London, United Kingdom; 5 School of Public Health, University of the Witwatersrand, Johannesburg, South Africa; 6 Malawi-Liverpool Wellcome Trust Clinical Research Programme, Blantyre, Malawi; 7 Institute for Global Health, University College London, London, United Kingdom

**Keywords:** tuberculosis, HIV, hospital, empirical treatment, mortality

## Abstract

**Background:**

Empirical tuberculosis (TB) treatment in human immunodeficiency virus (HIV)–positive inpatients is common and may undermine the impact of new diagnostics. We sought to describe empirical TB treatment and compare characteristics and outcomes with patients treated for TB after screening.

**Methods:**

This was a retrospective observational cohort study of HIV-positive inpatients treated empirically for TB prior to TB screening. Data on clinical characteristics, investigations, and outcomes were collected from medical records. Comparison cohorts with microbiologically confirmed or empirical TB treatment after TB screening with Xpert MTB/RIF and urine lipoarabinomannan assays were taken from South African Screening for Tuberculosis to Reduce AIDS-Related Mortality in Hospitalized Patients in Africa (STAMP) trial site. In-hospital mortality was compared using a competing-risks analysis adjusted for age, sex, and CD4 cell count.

**Results:**

Between January 2016 and September 2017, 100 patients excluded from STAMP were treated for TB empirically prior to TB screening. After enrollment in STAMP and TB screening, 240 of 1177 (20.4%) patients received TB treatment, of whom 123 had positive TB tests and 117 were treated empirically. Characteristics were similar among early empirically treated patients and those treated after TB screening. 50% of early empirical TB treatment was based on radiological investigations, 22% on cerebrospinal or pleural fluid testing, and 28% on clinical features alone. Only 11 of 100 empirically treated patients had subsequent microbiological confirmation. In-hospital mortality was lower in patients with microbiologically confirmed TB compared to those treated empirically (adjusted subdistribution hazard ratio, 0.5 [95% confidence interval, .3–.9).

**Conclusions:**

Empirical TB treatment remains common in severely ill HIV-positive inpatients. These patients may benefit from TB screening using existing rapid diagnostics, both to improve confirmation of TB disease and reduce overtreatment for TB.

## Teaser

Early empirical tuberculosis (TB) treatment in HIV-positive inpatients is common, with patients having similar characteristics but higher mortality than those with microbiological confirmation of TB. This group may benefit from rapid TB diagnostics improve confirmation of TB disease and reduce over-treatment.

Tuberculosis (TB) remains an important cause of admission to hospital and mortality in human immunodeficiency virus (HIV)–positive people living in sub-Saharan Africa [1], with postmortem studies suggesting that almost half of patients who died with TB in health facilities were undiagnosed and untreated at the time of death. Suboptimal diagnostics still drive this scenario despite substantial investment over the last 2 decades to improve diagnosis of HIV-associated TB, with development and implementation of Xpert MTB/RIF assay (Xpert), Xpert Ultra, and urinary lipoarabinomannan antigen (LAM) testing [2].

Sputum Xpert and urine LAM testing can increase TB diagnosis and treatment in hospitalized patients, with randomized clinical trial evidence for mortality impact in patients with more advanced disease [3, 4]. However, these trials excluded patients who were treated for TB within hours of their hospital admission, before screening and enrollment into the study was conducted. Given that HIV-associated TB often presents atypically, and even the best currently available diagnostics will not diagnose all patients with TB, “empirical” treatment (prior to testing or in the absence of a positive test) is a common and potentially life-saving approach. Indeed, it could be argued that requesting TB diagnostics is not warranted for patients for whom the decision to give TB treatment has already been made on the grounds of high pretest probability [5].

However, empirical TB treatment has adverse consequences, including high cost to both the patient and the health system, missing other diagnoses, drug toxicity and polypharmacy, delaying antiretroviral therapy (ART) initiation, and resource implications. Empirical TB treatment may also undermine the impact of new TB diagnostics [6]. Although World Health Organization (WHO) and national algorithms for TB diagnosis include empirical TB treatment, this should follow initial diagnostic testing for TB [7].

Few data exist describing HIV-positive patients receiving early empirical TB treatment during hospitalization. We therefore sought to describe the proportion of HIV-positive patients receiving empirical TB treatment early during hospital admission, their clinical characteristics, diagnostic modalities undertaken, and outcomes, compared to HIV-positive patients receiving TB treatment following TB screening as part of a trial and those not receiving TB treatment.

## METHODS

### Study Design and Procedures

This retrospective cohort study was conducted alongside the rapid urine-based Screening for Tuberculosis to Reduce AIDS-Related Mortality in Hospitalized Patients in Africa (STAMP) trial, Edendale Hospital site in KwaZulu-Natal, South Africa. Edendale Hospital is a regional, periurban hospital serving a population with high HIV and TB burden. The trial design has been described in detail elsewhere [3, 8]. In brief, HIV-positive adults (≥18 years old) admitted to medical wards were enrolled, irrespective of clinical presentation or symptoms, and randomized to 1 of 2 TB screening strategies (screening with sputum Xpert alone, or sputum Xpert plus urine LAM and Xpert testing). Patients were usually screened for trial enrollment on the day of admission (admission >48 hours prior to screening was an exclusion criteria).

Patients were excluded from STAMP if they had received TB treatment within the preceding 12 months, including TB treatment started after admission but prior to screening for the trial. TB screening was done within 24 hours of admission, and results were fed back to hospital clinicians. Clinical events during hospital admission were recorded, and the primary outcome of STAMP was mortality at 2 months. Routine diagnostics available to hospital clinicians included Xpert (sputum and nonrespiratory samples, on-site), chest radiography, ultrasound, and computed tomographic scanning. Mycobacterial culture was available, but samples had to be sent off-site, and this was not done routinely. Urine LAM testing was not available outside the STAMP trial during the study period, although clinicians could request urine Xpert. Non-TB diagnostics included HIV RNA load and cryptococcal antigen testing.

Patients were eligible for this study if they were excluded from the STAMP trial at screening due to TB treatment being commenced after admission but prior to trial screening procedures. Patients who were <18 years of age or HIV-negative were excluded. Patients were also excluded if TB treatment was commenced due to positive microbiological tests (ie, results available prior to TB treatment). Eligible patients were identified from STAMP screening databases from 1 January 2016 to 30 September 2017. Data on demographics, clinical characteristics, routine hematological and biochemical tests, TB diagnostic modalities, TB treatment, and vital status at discharge were extracted using custom-designed case report forms by retrospective review of medical records, prescription charts, and National Health Laboratory Service (NHLS) records. Patients for whom complete individual-level data linkage was not possible were excluded.

Patients enrolled in the STAMP trial between 1 January 2016 and 30 September 2017 at the Edendale Hospital site were also included as a comparison group. This STAMP patient group was further subdivided based on TB treatment: (1) patients not treated for TB during hospital admission; (2) confirmation of TB (eg, positive Xpert or urine LAM) and TB treatment started; and (3) no positive TB tests but TB treatment started empirically prior to hospital discharge.

### Ethical Considerations

The study was approved by the research ethics committee of the London School of Hygiene and Tropical Medicine (United Kingdom), and by the Biomedical Research Ethics Committee of the University of KwaZulu-Natal (South Africa). Patients in the STAMP trial provided written informed consent. A waiver of informed consent was granted for the early empirical TB cohort as only anonymized routinely collected data were used, and due to the retrospective nature of the study.

### Statistical Methods and Definitions

Study entry was defined as first documented encounter with inpatient services; time of TB treatment was decision to initiate, or initiation of, anti-TB therapy (whichever was sooner). Data on the main basis of TB treatment were based on medical records. TB diagnostic results were as documented in the medical records and/or NHLS record. Broad-spectrum antibiotics included at least 3 days of either ceftriaxone, co-trimoxazole (trimethoprim/sulfamethoxazole) at treatment dose, co-amoxiclav (amoxicillin-clavulanate potassium), azithromycin, piperacillin-tazobactam, imipenem, or cefotaxime. A positive WHO TB symptom screen was defined as 1 or more of cough, fever, weight loss, or night sweats. WHO danger signs were 1 or more of respiratory rate >30 breaths/minute, temperature >39°C, pulse >120 beats/minute, or inability to walk unaided. Microbiologically confirmed TB was defined as any positive mycobacterial culture, Xpert, or urinary LAM test. Mortality is reported during hospital admission (in-hospital mortality).

Proportions were compared using χ ^2^ tests, means using *t* tests, and medians using Wilcoxon rank-sum test as appropriate. In-hospital mortality risk was calculated from hospital admission, and compared between different groups receiving TB treatment (early empirical TB and those enrolled in the STAMP trial and started on TB treatment) using a competing risks model, with discharge alive from hospital as a competing risk, as this has been suggested as a more appropriate method of analyzing in-hospital mortality [9, 10]. Models were adjusted for age, sex, and CD4 cell count at admission a priori, and subdistribution hazard ratios (SHRs) were reported to describe associations with cumulative incidence accounting for competing risks [11]. To better understand associations with in-hospital mortality, patients were divided into 3 groups: (1) early empirical TB treatment (excluded from the STAMP trial); (2) enrolled in STAMP, started on TB treatment empirically with no positive TB tests; and (3) enrolled in STAMP with confirmation of TB (ie positive Xpert or urine LAM) and started on TB treatment. Sensitivity analyses were also conducted by including those undergoing early empirical TB treatment but with subsequent microbiological confirmation in group 3 instead of group 1. Analyses used Stata version 16 software and complied fully with Strengthening the Reporting of Observational Studies in Epidemiology (STROBE) and REporting of studies Conducted using Observational Routinely-collected Data (RECORD) guidance [12].

## RESULTS

**Figure 1.**
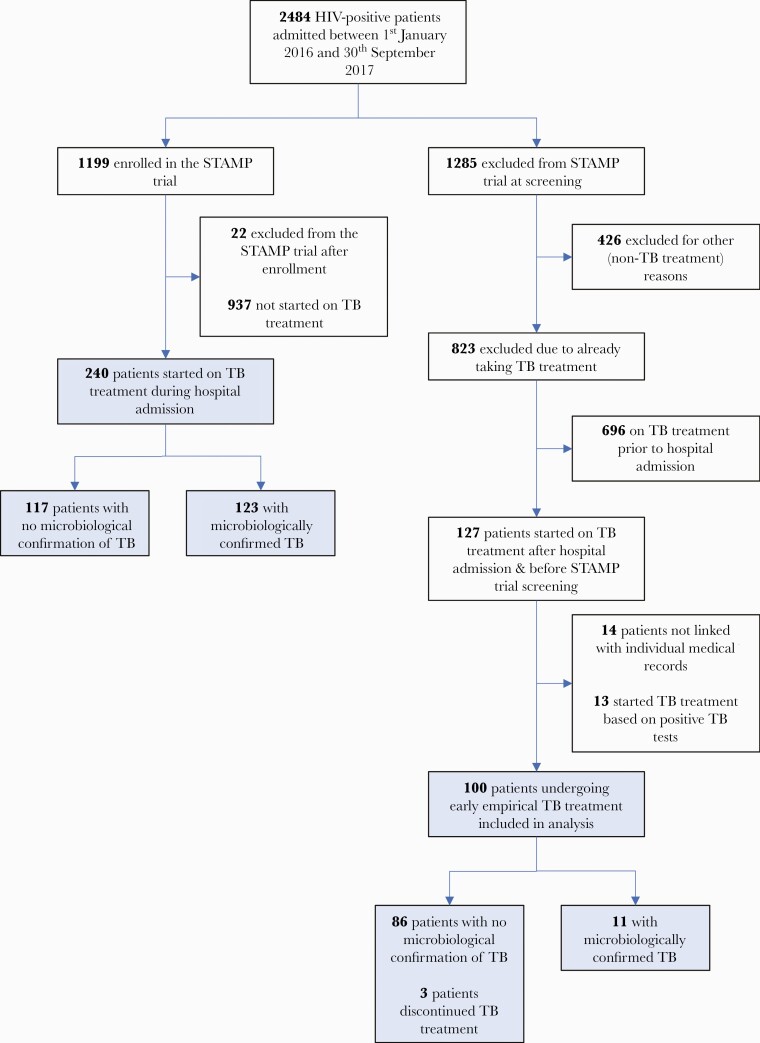
Flow of participants through the study. Abbreviations: HIV, human immunodeficiency virus; STAMP, Screening for Tuberculosis to Reduce AIDS-Related Mortality in Hospitalized Patients in Africa; TB, tuberculosis.

Between 1 January 2016 and 30 September 2017, 127 of 2484 (5.1%) HIV-positive patients admitted to medical wards received early presumptive TB treatment, and were therefore excluded from the STAMP trial (Figure 1). Of 113 of 127 (89.0%) with medical records available, 100 started TB treatment on criteria other than positive TB microbiological tests and were therefore included in the main analysis. During the same period, 240 (20.4%) patients enrolled in the STAMP trial were started on TB treatment during hospital admission, of whom 123 (51.3%) had positive TB tests.

### Baseline Characteristics

Patients receiving early empirical TB treatment were predominantly male. At least 94.0% had a positive WHO TB symptom screen and 39.0% had at least 1 WHO danger sign. Median C-reactive protein was 133 mg/L and patients had advanced immunosuppression at presentation (median CD4 count, 97 cells/µL). Baseline characteristics were similar to those of STAMP trial patients started on TB treatment after TB screening (both with and without positive TB tests), but early empirically treated patients were more severely immunosuppressed and more likely to have danger signs than STAMP trial patients not treated for TB (median CD4 count, 282 cells/ μL and 11.6% with WHO danger signs; Table 1).

**Table 1.** Baseline Characteristics and Outcomes

		Enrolled in STAMP Trial and Screened for TB		
Variable	Early Empirical TB Treatment	Treated for TB Without Positive Test (Empirical)	Treated for TB With Positive TB Test	Not Treated for TB
No. of patients	100	117	123	937
Age, y, mean (SD)	36.5 (9.8) (n = 100)	38.3 (10.4) (n = 117)	35.7 (10.3) (n = 123)	39.7 (12.0) (n = 937)
Sex				
Male	61 (61.0)	67 (57.3)	75 (61.0)	450 (48.0)
Female	39 (39.0)	50 (42.7)	48 (39.0)	487 (52.0)
HIV diagnosis				
New during admission	15 (15.0)	17 (14.5)	26 (21.1)	147 (15.7)
Diagnosed prior to admission	85 (85.0)	100 (85.5)	97 (78.9)	790 (84.3)
ART status				
Naive	37 (37.0)	30 (25.6)	43 (35.0)	253 (27.0)
Currently taking ART	50 (50.0)	77 (65.8)	68 (55.3)	647 (69.1)
Interrupted/stopped	13 (13.0)	10 (8.5)	12 (9.8)	37 (3.9)
Duration on ART, y, median (IQR)	0.4 (0.1–1.3) (n = 40)	0.9 (0.1–5.7) (n = 73)	0.9 (0.1–4.6) (n = 66)	2.7 (0.9–5.9) (n = 627)
Previous history of TB	20 (20.0)	44 (37.6)	36 (29.3)	335 (35.8)
Cough	61 (64)	87 (74.4)	99 (80.5)	489 (52.2)
Fever	44 (46)	84 (71.8)	88 (71.5)	509 (54.3)
Night sweats	41 (43)	67 (57.3)	71 (57.7)	373 (39.9)
Weight loss	68 (71)	96 (82.1)	118 (95.9)	638 (68.2)
Any WHO TB symptom	90 (94)	114 (97.4)	123 (100.0)	814 (86.9)
Any WHO danger sign	39 (39.0)	38 (32.5)	53 (43.1)	109 (11.6)
Hemoglobin, g/L, median (IQR)	102 (81–126)	99.0 (79.5–114.5) (n = 116)	88.0 (70.0–112.0) (n = 123)	117 (95–134) (n = 933)
CD4 count, cells/µL, median (IQR)	97 (32–265) (n = 85)	95.0 (29.0–250.0) (n = 117)	56.0 (18.0–171.0) (n = 123)	282 (113–489) (n = 932)
CRP, mg/L, median (IQR)	132.5 (54.0–228.0) (n = 72)	120.5 (50.0–232.5) (n = 112)	141.0 (84.0–201.0) (n = 119)	105.0 (72.0–174.0) (n = 25)
Creatinine, mmol/L, median (IQR)	85.0 (64.0–114.0) (n = 98)	83.0 (69.0–113.0) (n = 74)	101.0 (65.0–183.0) (n = 73)	82.5 (61.0–137.0) (n = 18)
TB treatment discontinued	3 (3.0)	2 (1.7)	0	…
Outcome at discharge				
Discharged alive	74 (74.0)	88 (75.2)	104 (85.2)	868 (92.7)
In-hospital death	26 (26.0)	29 (24.8)	18 (14.8)	68 (7.3)

Data represent absolute No. (%) unless otherwise indicated. Where data are missing, the numbers of included participants are shown in the table in parentheses. All variables are recorded at hospital admission. WHO TB symptoms are cough (of any duration), fever, night sweats, or weight loss. WHO danger signs are any of respiratory rate >30 breaths/minute, heart rate of >120 beats/minute, temperature >39°C, or being unable to walk unaided. Two patients are missing data on outcome (1 from the “treated for TB with positive test” group, and 1 from the “not treated for TB” group).

Abbreviations: ART, antiretroviral therapy; CRP, C-reactive protein; HIV, human immunodeficiency virus; IQR, interquartile range; SD, standard deviation; STAMP, Screening for Tuberculosis to Reduce AIDS-Related Mortality in Hospitalized Patients in Africa; TB, tuberculosis; WHO World Health Organization.

### TB Diagnosis and Treatment

In the early empirical group (n = 100), TB treatment was initiated within 6 hours for 35 (35.0%) patients, and within 24 hours in 73 (73.0%) patients. Median time to TB treatment was 15 hours (interquartile range, [IQR], 5–25 hours). In comparison, only 50 of 240 (20.8%) patients in the STAMP trial received TB treatment within 24 hours of admission, and median time to TB treatment was 24 hours (IQR, 24–72 hours).

The basis for starting early empirical TB treatment was results of radiology in 50 patients (50.0%), other investigations (cerebrospinal fluid [CSF] or pleural/ascitic fluid) in 22 (22.0%), and clinical features alone in 28 (28.0%) (Table 2). In total, 72 of 100 (72.0%) patients had a chest radiograph interpreted by clinicians as consistent with TB, of whom 42 (58.3%) were started on TB treatment based on this interpretation. Abnormal CSF protein and/or cell counts were the reason for starting TB treatment in 19.0% of patients. Twenty-four percent were treated for pulmonary TB and 25.0% for TB meningitis, and the remaining 51% were treated for extrapulmonary or disseminated TB (without meningitis). The decision to start TB treatment was made by senior clinicians for 66% of patients. Among patients screened for TB in the STAMP trial, TB treatment based only on clinical features or CSF was uncommon (8.3% and 4.6%, respectively; Table 2).

**Table 2.** Basis for Starting Tuberculosis Treatment and Tuberculosis Test Results

			Enrolled in STAMP Trial and Screened for TB			
	Early Empirical TB Treatment		Treated for TB Without Positive Test (Empirical)		Treated for TB With Positive Test	
Basis for Treatment and Results	No. (n = 100)	(%)	No. (n = 123)	(%)	No. (n = 117)	(%)
Basis for starting TB treatment						
Radiology	42	(42.0)	3	(2.4)	76	(65.0)
Plain radiograph	19	(19.0)	2	(1.6)	49	(41.9)
Ultrasound scan	3	(3.0)	1	(0.8)	19	(16.2)
CT scan	5	(5.0)	…	…	8	(6.8)
Microbiological TB test	0	…	120	(97.6)	…	…
Lumbar puncture and CSF	19	(19.0)	…	…	11	(9.4)
Pleural or ascitic fluid	3	(3.0)	…	…	10	(8.5)
Clinical features alone	28	(28.0)	…	…	20	(17.1)
TB test results^a^						
Sputum Xpert						
Negative	25	(73.5)	30	(27.8)	82	(100.0)
Positive	9	(26.5)	78	(72.2)	…	…
Sputum mycobacterial culture						
Negative	8	(88.9)	11	(91.7)	5	(100.0)
Positive	1	(11.1)	1	(8.3)	…	…
Nonrespiratory Xpert^b^						
Negative	30	(93.8)	51	(53.1)	62	(100.0)
Positive	2	(6.3)	45	(46.9)	…	…
Nonrespiratory mycobacterial culture						
Negative	1	(50.0)	8	(88.9)	5	(100.0)
Positive	1	(50.0)	1	(11.1)	…	…
Urine LAM^c^						
Negative	…	…	27	(33.3)	53	(100.0)
Positive	…	…	54	(66.7)	…	…

Data on basis for starting TB treatment are based on those reported by clinicians initiating TB treatment.

Abbreviations: CSF, cerebrospinal fluid; CT, computed tomography; LAM, lipoarabinomannan; STAMP, Screening for Tuberculosis to Reduce AIDS-Related Mortality in Hospitalized Patients in Africa; TB, tuberculosis; Xpert, Xpert MTB/RIF assay.

^a^Percentage denominator for TB test results is the number of patients for whom a test is performed. Test results available after decision to treat for TB in early empirical TB group. Patients may have >1 TB test.

^b^Nonrespiratory Xpert included CSF Xpert.

^c^Urine LAM was not available for the early empirical TB treatment group.

### Microbiological Investigations for TB

Among patients with early empirical TB treatment, only 34 (34.0%) had sputum samples and 32 (32.0%) had nonrespiratory samples tested with Xpert (Table 2). Forty-two percent of patients had no microbiological TB tests performed. Eleven (11.0%) had subsequent microbiological confirmation of TB; 8 patients had positive sputum Xpert, 1 had a positive nonrespiratory (lymph node aspirate) Xpert, 1 patient had both positive sputum and pleural fluid Xpert, and 1 patient subsequently had a positive nonrespiratory TB culture. Twenty-three patients had Xpert on CSF; all were negative. One patient was diagnosed with rifampicin resistance based on Xpert.

### Management and Outcomes

Eighty percent of early empirical TB patients received broad-spectrum antibiotics during admission. Additional opportunistic infections or HIV-associated conditions were diagnosed in 60 of 100 patients; 27 were diagnosed with concurrent bacterial infection, 8 with *Pneumocystis jirovecii* pneumonia, 10 with renal failure, 6 with toxoplasmosis, and 4 with cryptococcal meningitis (all cryptococcal antigen positive from CSF). Four (4.0%) patients had positive blood cultures; 3 isolated *Klebsiella pneumoniae* and 1 *Acinetobacter baumannii*. TB was the final diagnosis at discharge or death for 97 of 100 patients, with only 3 (3%) patients having TB treatment discontinued by clinicians due to alterative diagnoses. Of the 50 patients not taking ART at admission, 38 were recorded as starting ART, of whom 14 (36.8%) started within 2 weeks of admission, and 22 (57.9%) started within 8 weeks. Median CD4 count for those not initiating ART was 60 (IQR, 38–120; n = 9) and was similar to those initiating ART.

**Figure 2.**
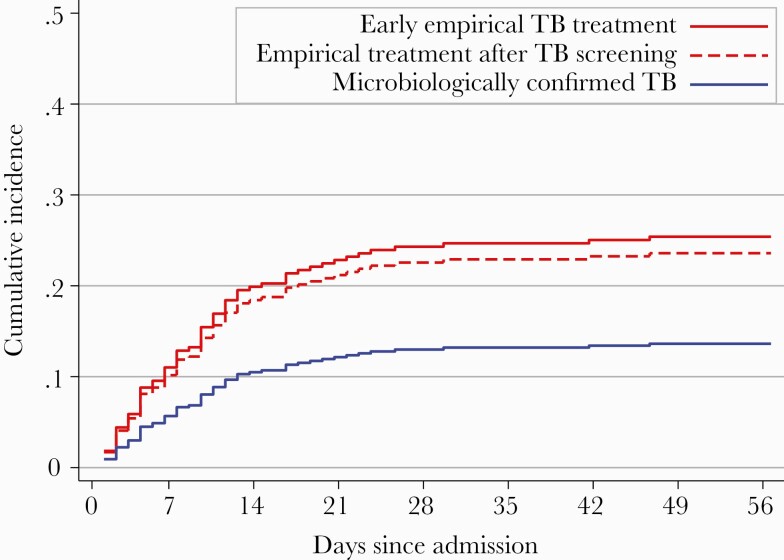
Cumulative incidence of in-hospital death by tuberculosis (TB) diagnosis type. Data represent cumulative incidence frequency of in-hospital death by TB treatment group, with hospital discharge as a competing risk. Model was adjusted for age, sex, and CD4 cell count at admission to hospital (n = 325). The “empirical treatment after TB screening” and “microbiologically confirmed” groups comprise patients enrolled in the Screening for Tuberculosis to Reduce AIDS-Related Mortality in Hospitalized Patients in Africa (STAMP) trial. The early empirical TB treatment group was excluded from STAMP trial due to their having already started TB treatment. In the model, the subdistribution hazard ratio for early empirical TB treatment is 1.09 (95% confidence interval, .61–1.9; ie, comparing the 2 red lines), and for microbiological confirmation is 0.52 (95% CI, .30–.91; *P* = .02; ie, comparing blue line with both red lines).

Twenty-six of 100 (26.0% [95% confidence interval {CI}, 18.2%–35.6%]) patients undergoing early empirical TB treatment died during hospital admission compared to 29 of 117 (24.8% [95% CI, 17.7%–33.5%]) of STAMP trial patients treated for TB with positive tests, 18 of 122 (14.8% [95% CI, 9.4%–22.1%]), and 68 of 936 (7.3% [95% CI, 5.8%–9.1%]) not treated for TB (Table 1). Median time to in-hospital death was 6 days (IQR, 3–11 days) in the early empirical group, and 9 days (IQR, 4–14 days) in the STAMP trial group, with few patients dying in the first 48 hours of admission (3.0% [3/100] and 3.8% [9/239] in the early empirical and STAMP trial groups, respectively). In models adjusted for age, sex, and CD4 cell count, in-hospital mortality was greater for patients treated for TB compared to those not treated for TB (adjusted SHR, 2.4 [95% CI, 1.6–3.4]), and those undergoing early empirical TB treatment compared to all STAMP trial patients treated for TB (adjusted SHR, 1.4 [95% CI, .9–2.4). Mortality was lower for patients with microbiological confirmation of TB than for patients treated empirically for TB in both the early empirical treatment group and STAMP trial patients (adjusted SHR, 0.5 [95% CI, .3–.9]; Figure 2). In-hospital mortality was similar in the early empirical treatment and empirically treated STAMP trial patients (adjusted SHR, 1.1 [95% CI, .6–1.9]; Figure 2). Sensitivity analyses including patients undergoing early empirical TB treatment but with subsequent microbiological confirmation of TB in the “microbiologically confirmed” group gave similar results (adjusted SHR, 0.5 [95% CI, .3–.9]).

## DISCUSSION

Our main findings are that early empirical TB treatment in HIV-positive patients admitted to hospital is common, and that baseline characteristics and outcomes were broadly similar to patients started on TB treatment empirically following TB screening in the STAMP trial. In this study, few patients treated empirically went on to have microbiological confirmation of TB disease, very few patients had TB treatment discontinued, and the risk of death during hospital admission was extremely high at 26%. Before and after adjusting for age, sex, and CD4 cell count, in-hospital mortality was substantially higher in empirically treated patients than those with microbiologically confirmed TB disease. Patients not receiving TB treatment had the lowest mortality.

Of all HIV-positive admissions, 5% received early empirical TB treatment, and of all patients started on TB treatment, approximately one-third underwent early empirical treatment prior to or without TB screening. This is a similar proportion to those starting TB treatment based on positive TB microbiological test results, demonstrating how common this practice is, and the high potential to impact evaluation of new rapid, more sensitive TB diagnostics [6]. The main drivers for early empirical TB treatment were clinical features (including patients being critically unwell, and failure to respond to antibiotics) and radiology, both of which have poor specificity for TB [13–16]. Suspected TB meningitis was also common in this cohort, although no cases were confirmed by Xpert or culture. Xpert Ultra on CSF and urine diagnostics (LAM and Xpert Ultra) seem to have diagnostic utility in this group [17–19]. Even following TB screening, clinicians still chose to treat for TB without microbiological confirmation in half of treated cases, highlighting that diagnostics still need improvement.

Data on empirical TB treatment in hospitalized HIV-positive patients undergoing routine clinical care are scarce. Two observational studies from sub-Saharan Africa suggested that empirical TB treatment may reduce mortality in smear-negative inpatients; however, these were both conducted prior to the availability of Xpert [20, 21]. An Ethiopian study of Xpert-negative hospitalized patients (21% HIV-positive) found substantial overtreatment but no differences in survival [22]. Data from outpatients have also reported widespread empirical TB treatment despite good availability of Xpert, but similar clinical outcomes, including mortality, between empirically treated and microbiologically confirmed TB patients [23, 24]. Furthermore, randomized trials have demonstrated that for outpatients, empirical TB treatment in advanced HIV is not superior to isoniazid preventive therapy, has no survival benefit compared to intensive TB screening, and results in significantly more adverse drug reactions [25–28].

While 2 randomized trials have demonstrated increased diagnosis and treatment of TB and mortality reductions with urine-based TB screening, patients undergoing empirical TB treatment very early during admission were excluded [3, 4]. We found that only 11% of early empirical treatment patients had microbiological confirmation of disease, and almost half did not have any Xpert or culture testing. Clinicians may not want to invest time in investigations that will not affect their management. However, this patient group would likely benefit from TB screening, for example with sputum Xpert and urine LAM assays (with or without urine Xpert), as it would increase the proportion of patients with TB microbiologically confirmed, including diagnosis of drug-resistant TB. Tuberculosis screening may also prevent the unnecessary prescription of TB treatment by providing more confidence in negative results. Empirical TB treatment without definitive diagnosis can cause significant harms beyond toxic effects of the drugs themselves, including economic costs to health care systems and to patients, missed drug resistance, delayed ART initiation, and increased morbidity and mortality from other diagnoses being missed [29].

In this study, 60% of patients were diagnosed with concurrent HIV-associated diagnoses. Our finding of higher mortality in empirically treated patients than among with microbiologically confirmed TB may be partially explained by higher prevalence of TB meningitis, untreated opportunistic infections (particularly central nervous system infections given high prevalence of abnormal CSF results), comorbidities, or HIV drug resistance [30]. This population could also benefit from enhanced diagnostics beyond TB coinfection alone. The early empirical TB treatment group also had a shorter median duration on ART, raising the higher risk of immune reconstitution inflammatory syndrome in this group. We also found delayed initiation of ART in patients undergoing early empirical treatment, with only 37% of those not already taking ART started within 2 weeks.

One argument to support empirical TB treatment is rapid treatment initiation, which has been hypothesized to potentially impact outcomes [31]. However, median time to TB treatment was similar in patients undergoing early empirical treatment and STAMP trial TB screening (15 hours vs 1 day, respectively), and few deaths occurred in the first 48 hours, suggesting that time gained by empirical TB treatment is likely to be negligible in the context of rapid diagnostics. However, it should be noted that neither Xpert nor culture are perfect reference standards, and the proportion of patients undergoing TB treatment without microbiological confirmation is broadly unchanged in the last decade [32]. Therefore, without more sensitive diagnostics, empirical TB treatment, especially in patients perceived to have the highest mortality risk, will continue. To improve empirical TB treatment decision-making we need a better understanding of the factors contributing to treatment thresholds, such as the local prevalence of TB, severity of illness, and perceptions of performance of diagnostics [33]. Other nonmicrobiological tools to help guide TB treatment decisions may be useful, for example, host response signatures [34]. It is also unclear if a lack of definitive microbiological TB diagnosis has an impact on TB treatment adherence.

The strengths of this study are being nested in a large clinical trial of TB screening, which ensured that all HIV-positive medical admissions were screened and accounted for, reducing the risk of bias, and providing a comparator group of patients with and without TB diagnoses. The study also reports routine clinical practice. There are also several limitations, including those inherent of the retrospective observational design. We were unable to locate medical records for 11% of patients, potentially introducing bias. There were also some missing data with regard to CD4 cell count data, and non-TB diagnoses were not confirmed beyond clinician diagnosis. Comparison groups between TB patients included and excluded from STAMP are not entirely consistent, as some patients undergoing early empirical TB treatment had subsequent microbiological confirmation, potentially resulting in bias. However, sensitivity analyses for mortality, which grouped these patients with “microbiologically confirmed” TB following TB screening in STAMP, gave almost identical results. Inherent differences between patients deemed to be too unwell or not suitable for TB screening prior to treatment may be hard to disentangle or adjust for, and data on patients’ overall clinical status were not available. Inpatients enrolled onto the STAMP trial were screened for TB by dedicated research nurses, and outcomes in this group may be different to patients screened during routine clinical care. There was no follow-up beyond discharge, so early postdischarge deaths would be unaccounted for. The study is also a single-site study. However, it does provide important data and insight into empirical TB treatment.

In conclusion, we show that early empirical TB treatment is common in severely ill HIV-positive patients presenting to hospital in a setting of high HIV and TB burden. These patients may well benefit from TB screening using newer, rapid and sensitive diagnostics such as urine LAM assays or Xpert Ultra, both to improve confirmation of TB disease and reduce overtreatment for TB. A better understanding of TB treatment decision making, including the impact of diagnostics, is warranted. This population is also a priority for better diagnostics for advanced HIV, which could potentially impact their high mortality risk.

## Notes

***Author contributions.*** D. W., K. F., E. L. C., and A. G. W. conceived the study. C. B., D. W., K. F., F. D. G., J. P., and A. G. W. designed the study. C. B., F. D. G., and J. P. contributed to data collection. C. B., A. G. W., and D. G. contributed to data analysis. C. B. and A. G. W. wrote the first draft. All authors contributed to revising the manuscript for intellectual content.

***Data availability.*** Data are not publicly available but are available on reasonable request to the authors, subject to relevant regulatory and ethical approval.

***Financial support.*** The Screening for TB to Reduce AIDS Related Mortality in Hospitalised Patients (STAMP) trial was funded by the Joint Global Health Trials Scheme of the UK Department of Health and Social Care; the Department for International Development; the Global Challenges Research Fund; the Medical Research Council; and the Wellcome Trust (number MR/M007375/1). A. G. W. has received a Royal College of Physicians London James Maxwell Grant Prophit Fellowship, and E. L. C. has received a Wellcome Trust Fellowship (number WT200901/Z/16/Z).

***Potential conflicts of interest.*** All authors: No reported conflicts of interest.
